# CoolBox: a flexible toolkit for visual analysis of genomics data

**DOI:** 10.1186/s12859-021-04408-w

**Published:** 2021-10-10

**Authors:** Weize Xu, Quan Zhong, Da Lin, Ya Zuo, Jinxia Dai, Guoliang Li, Gang Cao

**Affiliations:** 1 College of Veterinary Medicine, Huazhong Agricultural University, Wuhan, China; 2 State Key Laboratory of Agricultural Microbiology, Huazhong Agricultural University, Wuhan, China; 3 College of Informatics, Huazhong Agricultural University, Wuhan, China; 4 College of Bio-Medicine and Health, Huazhong Agricultural University, Wuhan, China; 5 National Key Laboratory of Crop Genetic Improvement, Huazhong Agricultural University, Wuhan, China; 6 Hubei Key Laboratory of Agricultural Bioinformatics, Hubei Engineering Technology Research Center of Agricultural Big Data, 3D Genomics Research Center, Huazhong Agricultural University, Wuhan, China

**Keywords:** Genomics, Visualization, Genome browser

## Abstract

**Background:**

Data visualization, especially the genome track plots, is crucial for genomics researchers to discover patterns in large-scale sequencing dataset. Although existing tools works well for producing a normal view of the input data, they are not convenient when users want to create customized data representations. Such gap between the visualization and data processing, prevents the users to uncover more hidden structure of the dataset.

**Results:**

We developed CoolBox—an open-source toolkit for visual analysis of genomics data. This user-friendly toolkit is highly compatible with the Python ecosystem and customizable with a well-designed user interface. It can be used in various visualization situations like a Swiss army knife. For example, to produce high-quality genome track plots or fetch commonly used genomic data files with a Python script or command line, to explore genomic data interactively within Jupyter environment or web browser. Moreover, owing to the highly extensible Application Programming Interface design, users can customize their own tracks without difficulty, which greatly facilitate analytical, comparative genomic data visualization tasks.

**Conclusions:**

CoolBox allows users to produce high-quality visualization plots and explore their data in a flexible, programmable and user-friendly way.

## Background

With the rapid development of Next-Generation Sequencing (NGS) technologies, more and more genomic assays have been developed to profile the genome from various aspects, such as RNA expression [1], protein-DNA binding [2], chromatin accessibility [3] and 3D structure [4, 5]. By integrating data from such types of different assays or the so-called multi-omics approach, biologists can comprehensively investigate genome dynamics during biological processes. This methodology has been successfully applied to many biological fields, such as neurological diseases [6], development of nervous system [7] and virus infection [8, 9]. Data visualization, especially the genome track like plots, are crucial for exploring or demonstrating some local or global properties of the genomics data.

Many visualization tools have been developed to meet these demands, and these tools can be classified into three categories: (1) Command-line plotting tool [10, 11], (2) Graphical User Interface(GUI) software [12], and (3) Web-based track browser [13–15]. In different situations, each kind of tools has its own advantages and limitations. As for command-line tools, they are convenient for bioinformaticians to produce plots or results easily but require Linux command line skills. GUI tools are friendly to people who are not skilled at programming and command line. Web-based browsers could share visualization results between colleagues. However, they are not efficient in transmission and have relative high latency between the websites and customers. Moreover, for program developers, GUI and web-based tools are not as convenient as command-line tools and plotting packages, which could be locally installed and easily called between stacks. Despite the above tools work well for providing an overview of the input genomic data. However, during actual scientific research, users need a detail comparative and analytical data visualization more than just the basic view of the data. For example, to visualize the differential contact interaction (DCI) of two Hi-C contact matrices [16] or predicted chromatin loops on the matrix [17]. In most cases, bioinformaticians work in programmatic and interactive environments like RStudio, IPython console and Jupyter notebook to complete the data analysis, algorithm development and visualization tasks. However, there is a gap between the data analysis ecosystem and the existing genomic data visualization tools. Researchers spend a lot of time on unnecessary stuffs like file format conversion and environment switching. Therefore, a versatile tool that fills the gap will significantly facilitate the genomics study.

To fill this gap, we developed CoolBox, a versatile toolkit for exploration-driven visualization of genomic data. It combines advantages of existing tools and is highly compatible with the Python scientific ecosystem, highly customizable, easy to use with intuitive interface design and simple installation procedure. It can be used in different scenarios: (1) Python script or another python package for data fetching and plotting; (2) Shell as a command-line plotting tool; (3) Jupyter notebook environment for data fetching, plotting, and exploration; and (4) Web application for exploration and demonstration within the web browser.

## Implementation

The plotting system of CoolBox is based on the matplotlib package. A part of the plotting code in the CoolBox is a fork from pyGenomeTracks package. [10] The data stored in bigWig, “.cool” and ”.hic” file format are loaded through pybbi (https://github.com/nvictus/pybbi), cooler [18] and straw [19] packages. Pairwise interaction data in Browser Extensible Data Paired-End (BEDPE) and Pairs format is indexed and randomly accessed using the pairix software (https://github.com/4dn-dcic/pairix). Other text-based genomic feature data format, like Browser Extensible Data (BED), Gene transfer format (GTF), and BedGraph is indexed and random accessed using the tabix [20] software. The widget panel in the GUI is implemented by using the ipywidgets package.

## Results and discussion

### Flexible and user-friendly API and CLI for producing high-quality genome track plots

**Fig. 1:**
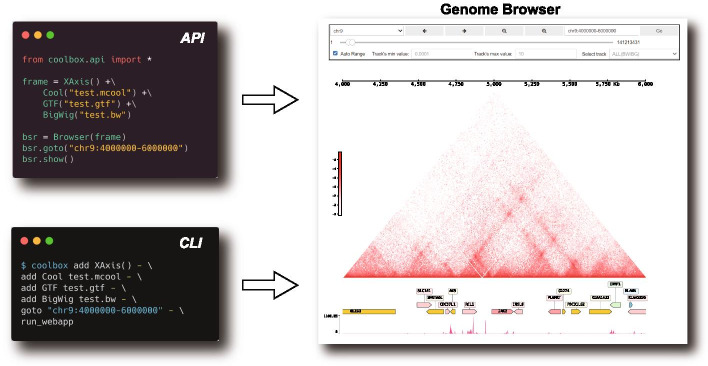
CoolBox has a clear and intuitive syntax to compose genome browser in both API and CLI mode. Inspired by the ggplot2 syntax, figures in CoolBox can be composed and adjusted (color, height, style etc.) from different tracks and features by using the ‘+‘ operator in API or ‘add‘ in CLI, almost every figure composed in the API mode has a paired CLI composing command that produces identical figures. This design facilitates bioinformaticians who work regularly in both environments

CoolBox provides an Application Programming Interface (API) for Python script or Jupyter environment as well as a Command Line Interface (CLI) for Shell. The interface design is inspired by the popular R package ggplot2 [21]. It allows users to compose their figures with highly intuitive syntax. In CoolBox, users can use the “+” operator in Python or “add” command in Shell to compose low-level track elements to a higher-level figure. For example, to compose track objects of various kinds of genomic data into a single frame and interactively review interested regions in genome browser with few lines of Python codes or Shell commands (Fig. 1).

**Fig. 2:**
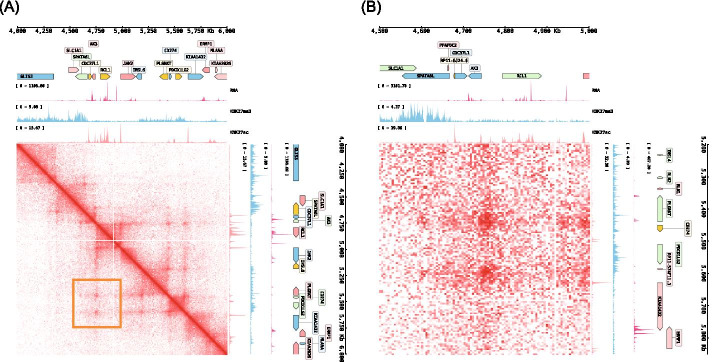
Joint (2d) view example, CoolBox can compose big figure which put frames around a center contact matrix. This allows to visualize the trans or cis remote (off-diagonal) contact matrix along with genome features. **A** Joint view on an on-diagonal region. **B** Joint view on an cis remote region, which shows the magnified detail of the orange box marked loop region that contains two chromatin loops in (**A**)

Besides the 1-dimensional viewing mode supported by most other visualization tools, CoolBox supports a joint-view mode that enables users to visualize trans or cis-remote regions in a Hi-C contact matrix (Fig. 2).

Most sets of commonly generated genomic assay data such as RNA-Seq, ChIP-Seq, ATAC-Seq, Hi-C, HiChIP [22] data which stored in BedGraph, bigwig [23], cool [18], .hic [19] and other file formats (see Table 1) can be visualized in CoolBox by different kinds of tracks. Most tracks’ features (color, height, style, etc.) can also be configured in the same way via the API or CLI. In the CoolBox plotting system, the plot contains not only a single layer. Users can put another layer (Coverage) upon the original plot to produce more comprehensive and high-quality figures. Furthermore, figures can be generated in different formats, including PNG, JPEG, PDF, and SVG. More details about the API and CLI are available in the online documents and user manual.

**Table 1:** A part of CoolBox builtin tracks for visualizing different kinds of genomics data formats

Track type	File format	Description
XAxis	None	Coordinate of the reference genome
Spacer	None	For add vertical space between two tracks
BigWig	.bigwig	Track for bigWig file, draw the histogram
BedGraph	.bedgraph	Track for BedGraph file, draw the histogram
BAM	.bam	BAM track for visualize the coverage or alignment
BED	.bed	For visualization genome annotation, like refSeq genes and chromatin states
GTF	.gtf	Track of GTF file, for visualize gene annotation
Arcs	.pairs, .bedpe	Show the chromosome interactions get from ChIA-PET, HiChIP or Hi-C loop data
HiCMat	.cool, .mcool, .hic	Show the chromosome contact matrix from Hi-C data
Virtual4C	.cool, .mcool, .hic	Virtual 4C track, using Hi-C data to mimic 4C
DiScore	.cool, .mcool, .hic	Directional index of Hi-C matrix for detecting TAD
InsuScore	.cool, .mcool, .hic	Insulation score of Hi-C matrix for inferring TAD borders
HiCDiff	.cool, .mcool, .hic	Show the difference between two contact matrix
Selfish	.cool, .mcool, .hic	Apply the selfish algorithm [16] on two contact matrices to detect differential contact interactions
SNP	.tsv	Track for show SNPs Manhattan plot

### Interactive exploration and reproducible analysis on genomic data

**Fig. 3:**
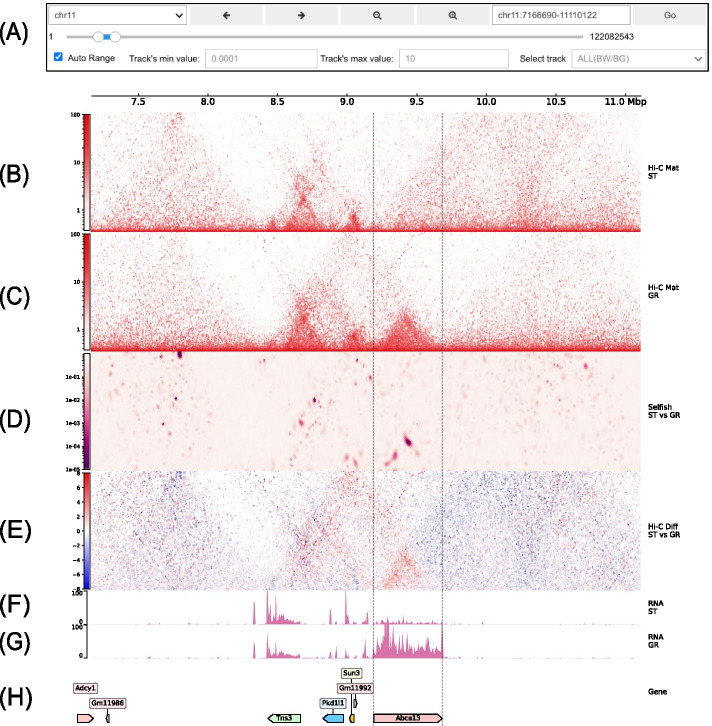
A CoolBox figure representing differential interactions of Hi-C contact matrices. Shown Hi-C and RNA-Seq data are produced from the process of hematopoietic differentiation [24]. Its clearly that there is a topological association domain (TAD) formation at the Abca13 gene region and its RNA expression is up-regulated at the same time after the differentiation. **A** The widgets panel of CoolBox browser, used for zooming, sliding, and locating the genome region. When moving to a new region, the figure draw bellow will be updated automatically. **B** Hi-C track of short-term hematopoietic stem cell (ST) shows the contact map of ST sample. The color bar indicates the normalized value of the contact map. **C** Hi-C track of granulocyte (GR). **D** Differential contact interaction result of the Selfish algorithm [16] on ST and GR Hi-C contact map. The color bar indicates the q-value (BH adjusted *p* value) produced from the DCI analysis. Darker color means this interaction has a lower q-value; that is to say, two contact maps are more diverse at this location. **E** Hi-C Diff track. It shows the difference between GR and ST’s z-score normalized contact matrices. The red region of the matrix indicates where GR has a more significant contact frequency compared to ST, and the opposite for blue areas. **F** BigWig track of ST RNA-Seq data, showing the RNA expression level of ST in this region. **G** BigWig track of GR RNA-Seq data. **H** A gene annotation track shows the corresponding genes within this genomic region

As shown in Fig. 3, CoolBox provides a GUI for interactive data visualization, by which users can explore different genomic regions by operating a simple widget panel and visualize the data within a specific region.

Besides, the data and the figures are bound together by Python objects. In this way, users can get the precise data of each track within the current view of the genomic region through the API. This design facilitates comparative visualization and statistical analysis. CoolBox is also a general genomic-file reading package. Data within a particular genome region can be retrieved in a short time, as almost all supported file formats can be indexed and randomly accessed.

Moreover, by leveraging the power of the Jupyter notebook, the visualization result and the entire process can be recorded in the notebook. It is convenient for sharing the visualization result and reproducing the whole analysis by other researchers.

### A testing and visualizing framework for new algorithm development

Owing to the user-friendly and highly extensible API design, users can implement their custom tracks without any difficulty, thus enabling seamless cooperation in Python-based algorithm development and scientific research. The algorithm developer can check and visualize the intermediate results produced by their algorithm and adjust parameters simultaneously. In addition, as CoolBox uses an object-oriented programming paradigm in its design, users can reuse each track’s codes by inheritance, including data extraction and drawing-related functions. In most cases, users only need to write algorithm-related core parts. The most tedious part including raw-data reading, preprocessing, and figure drawing are handed over to CoolBox through inheritance (see method section and user manual for implementation details). In this way, bioinformaticians can free themselves from those repetitive procedures and only focuses on the data post-processing.

**Fig. 4:**
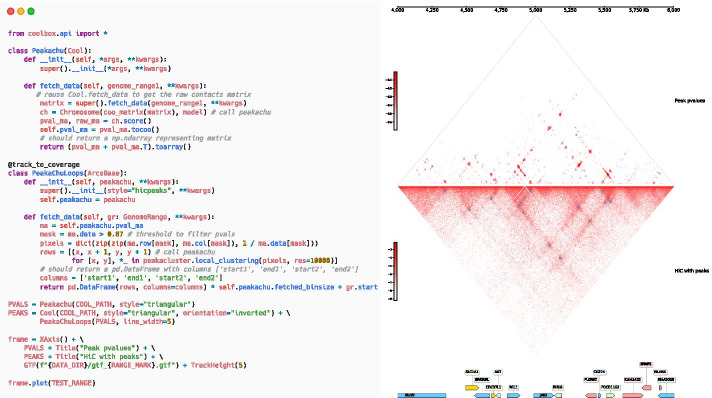
An example to define custom tracks that display Hi-C contact matrix along with peaks detected by Peakachu algorithm [17]. An example of peak prediction result is demonstrated in the right panel. The upper triangular matrix shows the peak p-value output by Peakachu algorithm. The predicted peaks drawn as blue squares upon the original matrix is shown in the lower triangular matrix. The Hi-C matrices and the peaks upon them will be automatically recomputed and updated after the change of genome region. The left panel is the full Runnable python codes used for generating the right panel. The custom track is implemented by following the same intuitive and clear design pattern as other built-in tracks: i.e., reusing the data fetching and plotting functionality as much as possible. For this figure, the functionality of fetching and plotting contact matrix with peaks are totally reused by inheriting Cool/ArcsBase track base class, and the rest of the codes merely calls the computing function of the peachachu package. After the track definition, we can see that the custom track is born to support being used in a ggplot2-like syntax with other tracks, and this capability is also valid in CLI and GUI mode

We demonstrate the advantages by implementing a track that visualizes the outputs of the Peakachu algorithm [17], which is a RandomForest based method for detecting loops in the Hi-C contact matrix. As depicted in Fig. 4, the main part of the whole track contains merely 20 lines of Python code. The data fetching and plotting functionality are fully reused by inheriting Cool/ArcsBase Track base class. Furthermore, the custom-defined track is empowered to be used in CLI, API, and browser mode in couple with other built-in tracks. More details include a reproducible code block and can be found in the online documents and user manual.

### Comparison with other existing visualization tools

As stated before, there is an urgent need for better visualization tools to accelerate the integration and mining of biological data. Therefore, more and more visualization tools have been developed in recent years. A comparison of features between CoolBox and these tools is listed in Table 2. Most of the visualization tools require a tedious installation process and are operated through the command line. Before visualization, the data needs to be preprocessed through specific steps, and then a static or interactive web interface is generated.

**Table 2:** Summary of genomic visualization tools

Tools	Programming language	API plot	CLI plot	Online access
CoolBox	Python	$\checkmark$	$\checkmark$	$\checkmark$
pyGenomeTracks	Python		$\checkmark$	
gcMapExplorer	Python	$\checkmark$		
HiCPlotter	Python		$\checkmark$	
HiGlass	Python, HTML, CSS, JS	$\checkmark$		$\checkmark$
YueLab Browser	HTML, CSS, JS			$\checkmark$
WashU Browser	HTML, CSS, JS			$\checkmark$
TADkit	HTML, CSS, JS			$\checkmark$
JuiceBox.js	HTML, CSS, JS			$\checkmark$
JuiceBox	Java			

GUI	Input	Installation	Customization	
Web and Jupyter	Raw data	Bioconda or PyPI	Python knowledge, very easy	
	Raw data	PyPI	Python knowledge, easy	
Local	Preprocessed data	PyPI	Python knowledge, easy	
	Preprocessed data	Manually install		
Web and Jupyter	Preprocessed data, via network	Docker	Web knowledge	
Web	Via network			
Web	Via network			
Web	Preprocessed data, via network	Manually install		
Web	Via network			
Local	Raw data	Download		

The visualization and data processing of most visualization tools are dissociated, which is not convenient for bioinformaticians whose routine works rely on Python-based scientific computation ecosystem. Except for the CLI mode supported by most visualization tools, the API that the CoolBox has been used internally and exposed follows the same design as the CLI, making switching between these two modes with no pain. More importantly, since the API in CoolBox combines computation and visualization, users can dynamically add different tracks or even custom tracks in the python notebook while processing raw data or developing new methods.

## Conclusion

CoolBox is a versatile toolkit for the visualization and exploration of multi-omics data in the Python ecosystem. It provides a user-friendly ggplot2-like syntax for composing various kinds of tracks in CLI, API, GUI and web browser mode. More importantly, its built on a highly extensible plotting system that allows users to implement their custom tracks without wasting time on data fetching and figure plotting procedures. Through the power of Jupyter notebook, it provides a convenient way for bioinformaticians to exploit it’s versatility for better personalized data manipulation and demonstration. It could also increase the reproducibility of genomic data visualization tasks as codes and figures are all organized into the same page.

## Availability and requirements

- Project name: CoolBox
- Project home page: https://github.com/GangCaoLab/CoolBox
- Operating system(s): Linux, macOS, Windows WSL
- Programming language: Python
- Other requirements: All software requirements are listed in https://github.com/GangCaoLab/CoolBox/blob/master/environment.yml
- License: GPLv3
- Any restrictions to use by non-academics: GPLv3 licensing restrictions apply.

## Data Availability

Sample data designed to demonstrate most features of the software is provided at https://github.com/GangCaoLab/CoolBox/tree/master/tests/test_data.

## Abbreviations

NGS: Next-generation sequencing
GUI: Graphical user interface
API: Application programming interface
CLI: Command line interface
